# Falciparum Malaria, Leptospirosis, and Vibrio mimicus Encephalitis in a Resilient Patient: A Remarkable Case of Triple Infection Survival

**DOI:** 10.7759/cureus.43879

**Published:** 2023-08-21

**Authors:** Keyur Saboo, Rinkle Gemnani, Sunil Kumar, Sourya Acharya, Preeti Mishra

**Affiliations:** 1 Department of Medicine, Jawaharlal Nehru Medical College, Datta Meghe Institute of Higher Education and Research, Wardha, IND; 2 Department of Pathology, Jawaharlal Nehru Medical College, Datta Meghe Institute of Higher Education and Research, Wardha, IND

**Keywords:** case report, triple strike, cerebral malaria, meningitis, leptospirosis, coinfections

## Abstract

Malaria is referred to as a "rainy season disease" and is brought on by *Plasmodium* species. *Leptospira interrogans*, a spirochete, cause zoonosis leptospirosis. It is pretty uncommon for both diseases to coinfect one another. Before assuming a fever is caused by a vector-borne disease, it is essential to first rule out other possible causes, regardless of the patient's risk factors. This case report demonstrates an unusual coinfection and how it manifests. The patient can avoid many deadly consequences with early detection and prompt treatment. There have been reports of coinfections between malaria and various infectious diseases, including dengue, hantavirus, and filariasis. Recently, a few case reports of coinfection with leptospirosis and malaria have also been published. Leptospirosis and malaria are both spreadable diseases that are prevalent throughout the world, particularly in the tropics. We discuss a case of coinfection with meningoencephalitis, leptospirosis, and malaria in a young male who required intensive care unit (ICU) care. It is difficult to distinguish between single infections and coinfections due to the wide variability in presentation, which may further confound the clinical features.

Furthermore, when a coinfection is present but has not yet been identified, the clinical course may worsen because there is no effective treatment. This case report demonstrates the uncommon coinfection appearance and related symptoms. The case study also examined the management of patients with leptospirosis, meningoencephalitis, and life-threatening malaria coinfections as well as the clinical course of such coinfections. A meningeal infection or inflammation that resembles both meningitis and encephalitis is referred to as meningoencephalitis.

## Introduction

Leptospirosis and other infectious illnesses, such as malaria, have overlapping geographic distributions. Many different diseases, including malaria, have been reported to coinfect people [1]. Only a few cases of leptospirosis and malaria coinfection have been reported. Clinical features are unreliable in differentiating between single and dual infections because of extremely similar clinical conditions. When patients with numerous infections present with considerable clinical symptoms, inadequate management may worsen their clinical outcome. This kind of coinfection commonly leads to inaccurate diagnosis, which worsens the situation and might even benefit the pathogens. The chance of coinfections may increase due to environmental exposure and the existence of similar pathogenic environmental microorganisms, which may also result in delayed or insufficient diagnosis [2].

Leptospirosis outbreaks are common in tropical and subtropical nations, and they are typically accompanied by natural catastrophes such as severe flooding and protracted periods of rain. Direct contact with water, soil, and vegetation polluted with pathogenic leptospires during water-related events is one of the well-known risk factors for leptospirosis. Agriculture, mining, maintaining sewage facilities, and taking part in military drills are a few of the occupations that place people at a greater risk of contracting an infection. This could present as mild, self-limiting febrile illness or can cause life-threatening illness with multi-organ dysfunction. Severe complications may lead to cerebral infections such as aseptic meningitis, meningoencephalitis, or cortical subarachnoid hemorrhage, which can present as agitation, psychosis, impaired consciousness, or coma.

India experiences high morbidity and mortality due to the deadly parasitic disease malaria. Early detection and thorough treatment are essential for the condition to be under control. An estimated 1.5 million confirmed cases are reported each year by the national vector-borne illness management program of the Indian government. Nearly half of these cases are caused by *Plasmodium falciparum*. If treatment is started as soon as possible, malaria can be cured. Delays in medical care could have fatal consequences. Treatment that is quick and effective is also necessary to control illness transmission. Malaria should be suspected in patients who reside in or have recently traveled to endemic areas. *Plasmodium vivax* takes 12-14 days to incubate, while *P. falciparum* takes 12-17 days [3,4]. Malaria can have neurological consequences such as seizures, psychosis, agitation, impaired consciousness, and even coma.

*Vibrio* species are a group of water bacteria that are often found in brackish or marine habitats. *Vibrio cholerae*, *Vibrio vulnificus*, and *Vibrio parahaemolyticus* are the three *Vibrio* species that are currently recognized to be responsible for the majority of human illnesses. *Vibrio mimicus* and *Vibrio cholerae* are genetically and phenotypically distinct from one another; however, *V. cholerae* can cause diarrheal disease because it secretes choleric toxin. Seafood, marine samples, human diarrheal stools, acute otitis brought on by exposure to seawater, and eggs in nests have all been linked to *Vibrio mimicus*. There have not yet been any cases of bacterial meningitis caused by *V. mimicus* reported in the most recent medical literature. At any age, *Vibrio* species are not known to cause meningitis. If this occurs, these pathogens are thought to infiltrate the central nervous system through an immune-compromised state and bloodstream infection [5]. Additionally, further research on the neurological manifestations of *Vibrio mimicus* is necessary. Here, we present a rare instance of a young person treated successfully with intravenous antibiotics, steroids, antimalarials, and other supportive drugs for bacterial meningoencephalitis brought on by *Vibrio mimicus*, falciparum malaria, and leptospirosis. A healthy male has been reported as the first potential case of meningoencephalitis caused by *Vibrio mimicus*.

## Case presentation

A 24-year-old male patient from the northeastern part of Maharashtra state who had previously been in good condition was admitted to the hospital with complaints of high-grade fever, severe headache, cola-colored urine, myalgia, and changed mental status. The patient has no previous history of tuberculosis, diabetes mellitus, or hypertension. The patient has no history of alcohol consumption in the past and no history of smoking.

Upon examination, the patient showed neck stiffness and other meningeal irritation symptoms. The patient's sensorium had been becoming worse for the past day, manifesting as diminished responsiveness to orders, decreased verbal output, and the inability to move his limbs. There was no prior history of unusual posture, rolling of the eyes, or mouth frothing, and no prior chest pain, palpitations, nausea, cold, or cough. The patient was feverish and in poor general health and had a blood pressure of 80/60 mmHg, a pulse rate of 120 beats per minute, and a room air oxygen saturation of 90%. The patient had neck stiffness, positive Brudzinski and Kernig signs, pupils that reacted to light by 4 millimeters, a Glasgow Coma Scale (GCS) score of E2V1M4, bilateral basal crepitations on auscultation, and drowsiness, according to a central nervous system examination. The patient's condition deteriorated on the next day, with increased drowsiness and a GCS score of E1V1M3, which required intubation.

**Figure 1:**
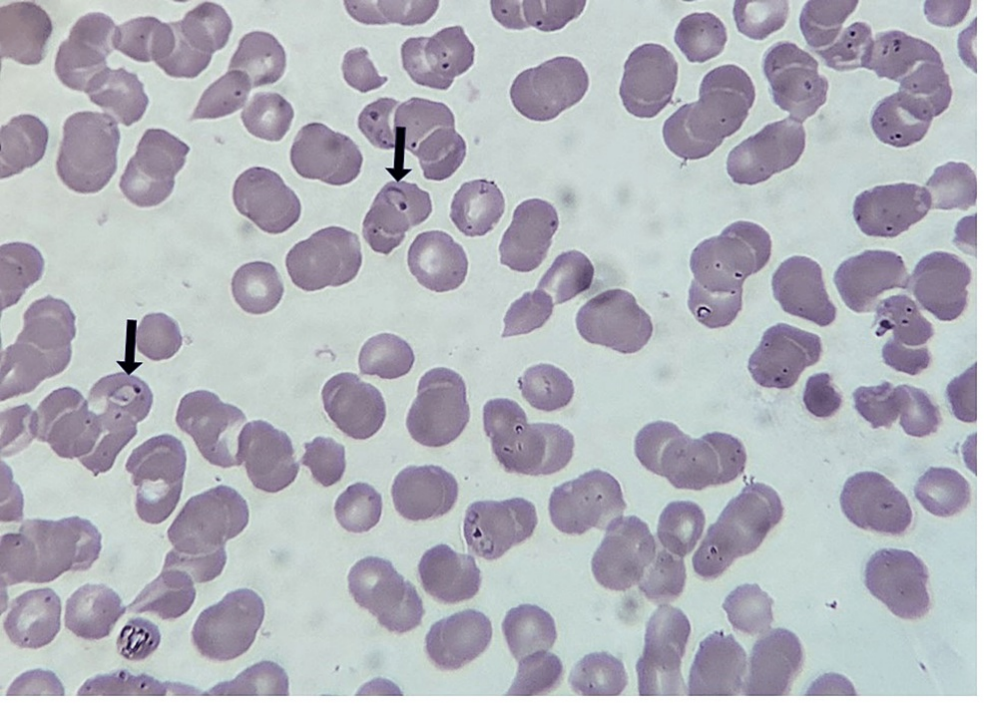
Plasmodium rings on a thick blood smear (black arrows).

**Figure 2:**
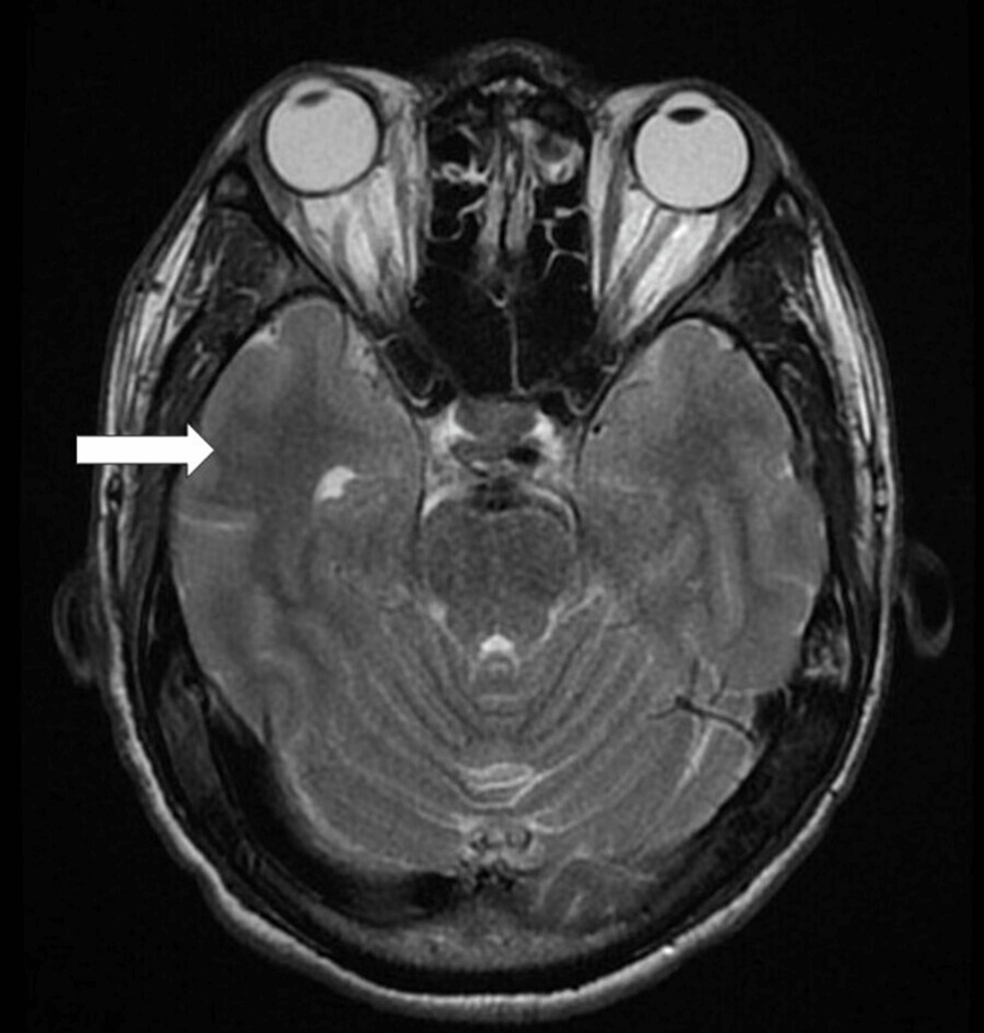
Patient's magnetic resonance imaging scan revealing the presence of encephalitis (white arrow).

**Figure 3:**
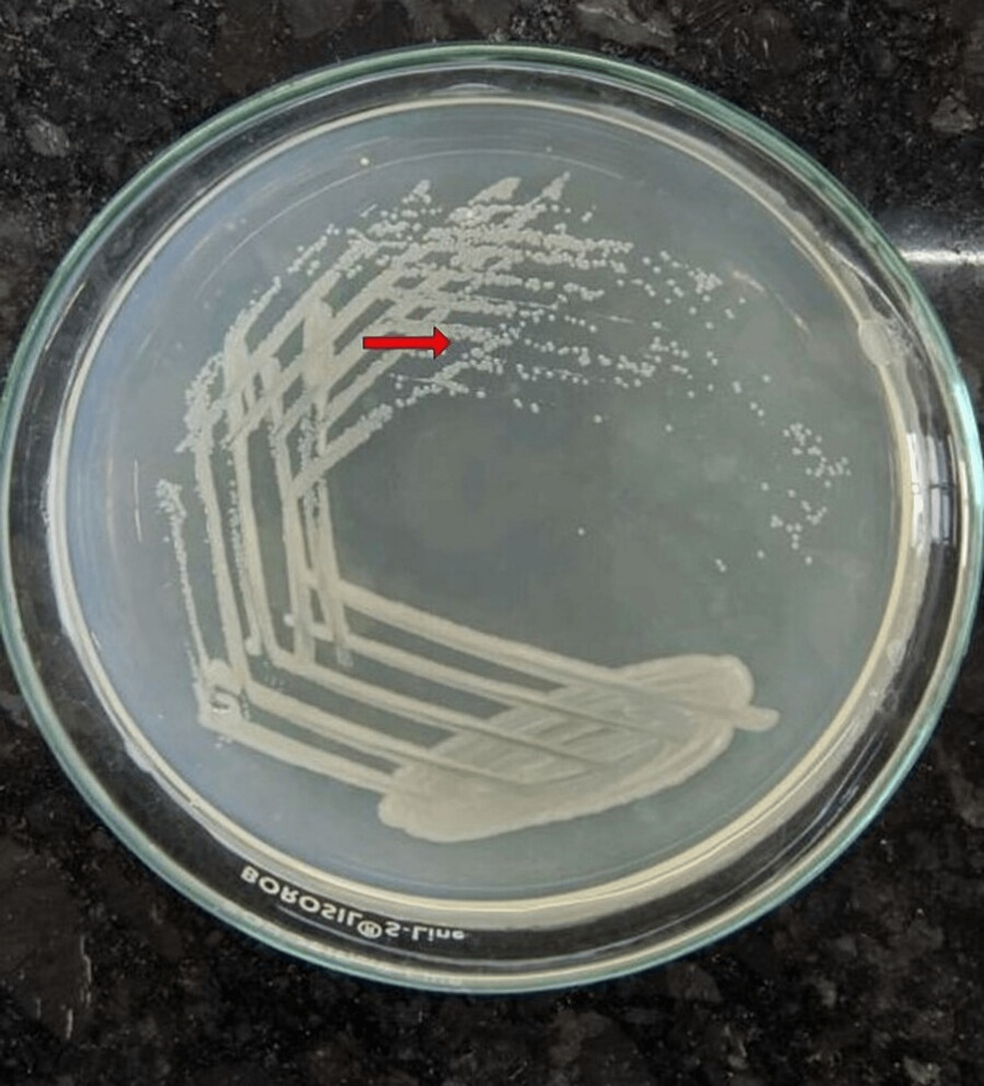
Growth of Vibrio mimicus in cerebrospinal fluid on nutrient agar medium (red arrow).

Laboratory tests showed severe anemia, thrombocytopenia, and increased liver enzymes (Table 1). Investigations were conducted after a syndromic examination, and falciparum parasitemia was found. Falciparum ring formations were detected in a thick blood smear (Figure 1). The results of the blood, sputum, and urine cultures were negative. The patient's human immunodeficiency virus (HIV) and hepatitis status were negative. He worked in a paddy field where rodents were occasionally observed; therefore, leptospirosis was suspected. Leptospira microscopic agglutination test (MAT) was requested, and the diagnosis was verified. Acute meningoencephalitis may be present as indicated by the results of the brain magnetic resonance imaging (MRI) (Figure 2). Cerebrospinal fluid (CSF) tests confirmed bacterial meningitis following lumbar puncture (LP) (Table 2). *Vibrio mimicus* was eventually grown in CSF cultures (Figure 3).

**Table 1:** Investigation profile of the patient at the time of admission.

Investigations	Patient	Reference values
Hemoglobin	5.7 g/dL	13-17 g/dL
Total leukocyte count	12,300/dL	4,000-11,000/dL
Platelet count	57,000/dL	150,000-400,000/dL
Serum creatinine	0.7 mg/dL	0.5-1.2 mg/dL
Albumin	2.1 g/dL	3.5-5 g/dL
Aspartate aminotransferase	116 U/L	<50 U/L
Alanine aminotransferase	50 U/L	17-59 U/L
Total bilirubin	2.5 mg/dL	0.2-1.3 mg/dL
C-reactive protein	103 mg/dL	<6 mg/dL
Erythrocyte sedimentation rate	32 mm/hour	0-20 mm/hour

**Table 2:** Cerebrospinal fluid analysis on presentation. CSF: cerebrospinal fluid

Parameters	Reference range	Patient sample
Red blood cell count	<2/uL	46/uL (high)
Total nucleated cells	<5/uL	126/uL (high)
Color, CSF	Clear	Yellow
Glucose, CSF	40-70 mg/dL	<22 mg/dL (low)
Protein, CSF	15-45 mg/dL	124 mg/dL (high)

The patient was empirically started on injections of artesunate 120 mg at 0 hours, 12 hours, and 24 hours, followed by 120 mg once a day for seven days, along with other supportive treatments such injections of penicillin 1.5 million units IV six hourly, ceftriaxone 2 gm twice daily, injection of doxycycline 100 mg twice daily for seven days, and tablets of primaquine 15 mg twice daily. Over the course of the next three days, as the hemolysis gradually decreased, the patient's sensorium got better, and he became afebrile. Infections are frequent and can go undiagnosed in the tropics. Failure to treat any coinfections could have unfavorable effects. Following seven days of intravenous antiparasitic treatment, the patient was discharged. Following discharge, the patient was told to check on their family. After a month, the patient's follow-up visit revealed good progress.

## Discussion

It is estimated that there are 500 million cases of malaria worldwide, with 2-3 million instances of severe malaria and 1.1 million fatalities annually. Infections such as leptospirosis are frequent in tropical regions. Large epidemics are observed following periods of severe rain. In some parts of China, Southeast Asia, Africa, and South and Central America, the disease is endemic. In Kerala, Tamil Nadu, and the Andaman Islands of India, leptospirosis is endemic. Coinfections are widespread since leptospirosis and malaria are both widely found in the tropics. In a recent study, Singhsilarak et al. discovered that 7.7% of adult patients with falciparum malaria had leptospirosis coinfections in their serum [6]. However, only 15 cases of coinfection with leptospirosis and malaria (eight from India and seven from Thailand) have been documented clinically in the literature. They are under-recognized since the majority of infections are anicteric, remit on their own, overlap with other febrile illnesses in terms of clinical characteristics, and are difficult to diagnose because leptospiral assay kits are not readily available. Leptospirosis causes pulmonary lesions that are predominantly hemorrhagic and have a 20%-70% incidence. The degree of involvement may be little or substantial and deadly, and it may not be related to the existence of icterus [2].

Significant organ sequestration may cause endothelial dysfunction and blockage in patients with severe falciparum malaria. Endothelial dysfunction may lead to micro-thrombosis and vasospasm, which can result in decreased blood flow to the organs and a rise in lactic acidosis. The differential diagnosis for a patient with fever, renal failure, and jaundice include severe leptospirosis, severe malaria, enteric fever, hantavirus, viral hepatitis with fulminant liver failure, and scrub typhus. Based on the local epidemiology, hemolysis, and lung injury, the differential diagnosis for this patient was restricted to viral hepatitis, specifically, hepatitis E virus-related viral hepatitis, leptospirosis, typhoid, and hemolysis. Third-generation cephalosporin, doxycycline, and antimalarial drugs are first warranted for use in coinfection situations since they treat the majority of the causing agents, including a malarial parasite, leptospirosis, rickettsia, and salmonella. However, a delayed start to guided therapy and potentially avoidable excess mortality occurs when acute leptospirosis coinfection is not recognized. As a result, the proper application of antibiotics, under the direction of clinical judgment, is crucial and cannot be replaced by adjunct therapies [7].

Leptospirosis can be diagnosed using various methods, including the microscopic agglutination test (MAT), slide agglutination test (SAT), and IgM enzyme-linked immunosorbent assay (ELISA). The MAT, previously considered the gold standard, has now been replaced by SAT and IgM ELISA, which are more precise and straightforward. MAT's complexity, including the need for dark-field microscopy and cultures of different serovars, has made it difficult to use in smaller laboratories. The ELISA IgM and SAT tests, which can diagnose active leptospirosis from a single sample, have rapidly and precisely identified IgM antibodies. Additionally, the IgM ELISA test can detect a positive result as early as two days after infection, making an early diagnosis easier. It has been found to be 93% specific and 100% sensitive in some studies [7].

Water bacteria known as *Vibrio* species are typically found in brackish or marine environments. *Vibrio cholerae*, *Vibrio vulnificus*, and *Vibrio parahaemolyticus* are the three *Vibrio* species that are most frequently linked to human infections. The two main categories of human illnesses are cholera and non-cholera infections. The cause of cholera, which is very severe diarrhea, is *Vibrio cholerae*. Vibriosis, a series of illnesses with varying clinical symptoms depending on the pathogen species, route of infection, and host susceptibility, is caused by non-cholera *Vibrio* species such as *Vibrio vulnificus* and *Vibrio parahaemolyticus*. Since its discovery in 1983, *Vibrio mimicus* has come to be known as a rare cause of gastroenteritis following recent seafood consumption as well as acute otitis media following contact with seawater. Although they are more frequently seen in marine habitats, some species, including *Vibrio mimicus*, may also survive in freshwater [8].

This is the first instance of *Vibrio mimicus*-related meningitis that has been documented in the literature, to our knowledge. The most likely location for our patient to get an infection was on the ground. The patient was a student who had to work in the mud because his father was a farmer. Given that, he must have come into contact with the microorganisms that led to his bacteremia and the meningitis that followed through the soil. Early detection of meningoencephalitis is necessary for efficient and targeted treatment, yet substantial morbidity and mortality still occur as a result. In many cases, the encephalitis begins as unilateral but later spreads to both sides, becomes bilateral, and has an unequal impact on the temporal and contralateral lobes.

## Conclusions

It is important to keep a high level of suspicion for coinfections, although they are rare. Delayed diagnosis can lead to higher morbidity and death rates. If a patient presents with severe malaria, fever, thrombocytopenia, altered liver function tests, and altered renal function tests, empirical treatment with third-generation cephalosporin, doxycycline, and antimalarial medications should be considered. This treatment covers most of the causative agents, such as malarial parasites, leptospira, rickettsia, and salmonella in cases of coinfection. Leptospirosis can rapidly deteriorate in its severe form, so it is important to treat it with caution.
